# Atorvastatin improves cisplatin sensitivity through modulation of cholesteryl ester homeostasis in breast cancer cells

**DOI:** 10.1007/s12672-022-00598-8

**Published:** 2022-12-08

**Authors:** Diandra Zipinotti dos Santos, Isabella dos Santos Guimaraes, Mariam F. Hakeem-Sanni, Blake J. Cochran, Kerry-Anne Rye, Thomas Grewal, Andrew J. Hoy, Leticia B. A. Rangel

**Affiliations:** 1 Biotechnology Program/RENORBIO, Health Sciences Center, Universidade Federal do Espírito Santo, Vitoria, ES Brazil; 2 Division of Clinical Research, Research Center, Instituto Nacional do Câncer, Rio de Janeiro, Brazil; 3 School of Medical Sciences, Charles Perkins Centre, Faculty of Medicine and Health, The University of Sydney, Sydney, NSW Australia; 4 School of Medical Sciences, Faculty of Medicine, UNSW, Sydney, NSW Australia; 5 School of Pharmacy, Faculty of Medicine and Health, The University of Sydney, Sydney, NSW Australia; 6 Biochemistry Program, Health Sciences Center, Universidade Federal do Espirito Santo, Vitoria, ES Brazil; 7 Department of Pharmaceutical Sciences, Universidade Federal do Espírito Santo, Vitória, Brazil

**Keywords:** Cisplatin, Atorvastatin, Cholesteryl ester, ACAT-1, Chemoresistance, Breast cancer

## Abstract

**Background:**

Acquired treatment resistance is a significant problem in breast cancer management, and alterations in lipid metabolism have been proposed to contribute to the development of drug resistance as well as other aspects of tumor progression. The present study aimed to identify the role of cholesterol metabolism in MCF-7 and MDA-MB-231 breast cancer cell response to cisplatin (CDDP) treatment in the acute setting and in a model of CDDP resistance.

**Methods:**

MCF-7 (luminal A), MDA-MB-231 (triple-negative) and CDDP-resistant MDA-MB-231 (MDACR) cell lines were grown in the presence or absence of CDDP in combination with atorvastatin (ATV), lipid depletion or low-density lipoprotein loading and were analyzed by a variety of biochemical and radiometric techniques.

**Results:**

Co-administration of CDDP and ATV strongly reduced cell proliferation and viability to a greater extent than CDDP alone, especially in MDA-MB-231 cells. These findings were associated with reduced cholesteryl ester synthesis and storage in MDA-MB-231 cells. In MDACR cells, acetyl-CoA acetyltransferase 1 (ACAT-1) was upregulated compared to naïve MDA-MB-231 cells and ATV treatment restored CDDP sensitivity, suggesting that aberrant ACAT-1 expression and associated changes in cholesterol metabolism contribute to CDDP resistance in MDA-MB-231 cells.

**Conclusion:**

These findings indicate that the elevated susceptibility of MDA-MB-231 cells to co-administration of CDDP and ATV, is associated with an increased reliance on cholesteryl ester availability. Our data from these cell culture-based studies identifies altered cholesterol homeostasis as an adaptive response to CDDP treatment that contributes to aggressiveness and chemotherapy resistance.

**Supplementary Information:**

The online version contains supplementary material available at 10.1007/s12672-022-00598-8.

## Introduction

Breast cancer (BC) is a heterogeneous disease composed of five major biological subtypes based on microarray gene classifications [1]. Many of these subtypes can be successfully treated; however, despite well-established molecular targets in most BC subtypes, the development of drug resistance still occurs. Another major challenge is the treatment of triple-negative BC (TNBC) due to the lack of the druggable targets estrogen receptor, progesterone receptor and human epidermal growth factor receptor 2. As such, TNBC, as well as any other BCs that do not respond well to inhibitors of the abovementioned targets, are commonly treated with non-specific chemotherapy, like cisplatin (CDDP) [2]. However, these treatments also develop resistance and result in disease relapse, leading to a poor prognosis of drug-resistant BCs, particularly TNBC [3]. Hence, a better understanding of BC biology, including TNBC, and the mechanisms associated with treatment resistance is still required to improve patient outcomes.

Anomalous lipid metabolism and signaling have been implicated in oncogenesis [4], and changes in tumor cholesterol metabolism are often associated with disease progression [5]. In general, cholesterol homeostasis is maintained by a balance between *de novo* biosynthesis, mainly regulated by 3-hydroxy-3-methyl-glutaryl-coenzyme A reductase (HMGCR), the uptake of exogenous low-density lipoprotein (LDL)-cholesterol by the LDL-receptor (LDLR), and esterification of endogenous as well as LDL-derived cholesterol by acetyl-CoA acetyltransferase 1 (ACAT-1). Notably, many cancers exhibit increased LDLR expression and activity, LDL uptake, and upregulated cholesterol synthesis and esterification, as well as loss of fundamental homeostatic control of cholesterol metabolism whereby the negative feedback relationship between *de novo* synthesis and uptake via LDLR is dysregulated [6, 7]. At the whole-body level, elevated LDL has been associated with poor disease prognosis [8] and increased levels of circulating 27-hydroxycholesterol, a common oxysterol derived from cholesterol, correlated with estrogen receptor (ER)-positive BC tumor growth and metastasis [9]. In contrast, statin use has been associated with lower BC recurrence and cancer-specific mortality [10, 11]. In fact, targeting key cholesterol metabolic pathways in cancer cells slowed proliferation [6], migration/invasion, and tumor size in mouse models of BC [6, 7, 12]. Altered cholesterol metabolism has also been linked to the development of treatment resistance [5, 13]. Evidence supporting this include reports that changes in cholesterol homeostasis coincide with the development of resistance to tamoxifen in luminal A BC [14] and in leukemic cell lines [15, 16]. However, the role of cholesterol metabolism in cancer cells, in particular other BC subtypes, and its potential contribution to treatment resistance against non-specific chemotherapy is less well described. As such, a better understanding of how cells metabolize cholesterol, and its functionalities, may lead to effective therapeutic strategies.

Since CDDP is a commonly used drug in BC treatment, our study explored aspects of CE metabolism as potential adaptive mechanisms in response to CDDP treatment in BC cell lines. Further, we evaluated the efficacy of targeting cholesterol availability to improve CDDP sensitivity, both acutely and in CDDP-resistant cells. We hypothesize that changes in CE homeostasis are an adaptive response to CDDP treatment and that cholesterol-lowering using statins improves CDDP sensitivity and overcomes CDDP resistance.

## Materials and methods

### Cell culture and reagents

MDA-MB-231 and MCF-7 cells were purchased from ATCC. A CDDP-resistant cell line, MDACR, was generated in-house from its parental lineage, MDA-MB-231 and selected for progressive resistance to CDDP following a protocol previously described [17]. Cells were grown in high glucose DMEM (Invitrogen) supplemented with 10% (v/v) Fetal Bovine Serum (FBS) (Invitrogen), 10,000 U/ml penicillin/streptomycin (Sigma-Aldrich) at 5% CO_2_, 37 °C. Lipoprotein-deficient fetal bovine serum (LPDS) was prepared by ultracentrifugation as described [18]. LDL was isolated from donated, pooled blood samples from normal healthy donors (obtained from the Red Cross, Sydney, Australia) by three sequential density gradient ultracentrifugations in KBr gradients (density 1.019–1.055 g/ml). All experimental protocols for the use of blood products purchased from the Red Cross (Material Supply Agreement no: 19-07NSW-1) for the isolation of plasma lipoproteins were approved by the local ethics committee of the University of New South Wales (HC190432) in accordance with the National Health and Medical Research Council’s (NHMRC) National Statement on Ethical Conduct in Human Research (2007). Pure atorvastatin calcium salt (ATV) was kindly donated by Dr. Paulo Alexandre Palacio (O2 Manipulação, Brazil). CDDP was purchased from Cayman Chemical. ATV was diluted in DMSO (final concentration ≤ 1% v/v) (Sigma-Aldrich) and CDDP in phosphate-buffered saline (PBS).

### Cell viability

Cell viability was evaluated by the thiazolyl blue tetrazolium blue (MTT) assay as described previously [17, 19]. To address the impact of lipid depletion or LDL loading on cell viability, 0.5 × 10^5^ cells/mL were plated and incubated overnight, the seeding medium was removed and replaced with fresh medium containing drugs of interest and 10% FBS or 10% LPDS for 48 h. Following, 50 µg/ml LDL were added to LPDS-preincubated cells for an additional 24 h to evaluate the recovery of cell viability in LDL-cholesterol-enriched conditions. The percent cell viability relative to the control (untreated cells) contained only drug solvent was calculated using the following equation:

$$\% {\text{Cell}}\,{\text{viability}} = ({\text{Abs}}\,{\text{drug}} - {\text{treated}}\,{\text{cells}}/{\text{Abs}}\,{\text{vehicle}} - {\text{treated}}\,{\text{cells}})\, \times \,100$$

The half maximal inhibitory concentration (IC_50_) of CDDP and ATV was determined as the drug concentration that resulted in 50% cell growth inhibition, as compared with the growth of the control cells, following 48 or 72 h exposure to the drugs.

### Cell confluency

The IncuCyte^®^ S3 Live-Cell Analysis System was used to measure cell confluency according to the manufacturer’s instructions as described [19]. Cells were seeded at 0.5 × 10^5^ per well in 96-well plates and incubated overnight at 37 °C in a humidified atmosphere (5% CO_2_). The seeding medium was replaced the next day with fresh medium containing different concentrations of the drugs of interest or medium with 10% FBS, 10% LDPS or 10% LPDS + 50 mg/ml LDL. Cell confluency was calculated as percent relative to Control at 48 and 72 h for each treatment.

### Determination of cellular CE levels

Cellular CE levels were measured using the Amplex^®^ Red Cholesterol Assay Kit (Invitrogen) as described [19]. Briefly, cells were harvested, washed with PBS and an aliquot used for protein quantitation by BCA protein assay (Thermo Fisher Scientific). Cellular lipids were extracted following the Folch extraction method [20], and samples were prepared according to the manufacturer’s protocol.

### Determination of cholesterol esterification

Cells were incubated in high glucose DMEM containing 0.5 mM oleate, 2% w/v bovine serum albumin (BSA; Sigma) and 1 µCi/ml [9,10-^3^ H]-oleate (Perkin Elmer) for 4 h at 5% CO_2_, 37 °C. Cells were harvested and washed with PBS, and lipids extracted to determine ^3^ H-oleate incorporation into cholesteryl esters using the Folch extraction method (21). Intracellular lipids were resuspended in a chloroform/methanol (2:1) mixture, spiked with cholesterol and cholesteryl oleate (Sigma), and separated by thin-layer chromatography, using hexane/isopropyl ether/acetic acid (60:40:3) as the solvent system. ^3^ H activity in CE bands was determined by liquid scintillation counting.

### Immunoblotting

Protein extraction from cultured cells was performed as described previously [21]. Samples (30 µg protein/sample) were separated on 10% SDS-PAGE and transferred to polyvinylidene fluoride (PVDF) membranes (Merck). Membranes were blocked for 1 h at room temperature with 5% BSA and incubated overnight at 4 °C with primary antibodies against HMGCR (rabbit anti-HMGCR; ab174830; 1:1,000; Abcam), ACAT-1 (mouse anti-ACAT-1; sc-69,836; 1:1,000; Santa Cruz Biotechnology), LDLR (rabbit anti‐LDLR; ab52818, 1:500; Abcam), and 14-3-3 (rabbit anti-14-3-3, #8312S, 1:1000, Cell Signaling Technology). Membranes were washed, incubated for 1 h with the appropriate secondary antibodies at room temperature, and the immunoblots were developed using enhanced chemiluminescence reagent (ECL plus; Merck). Bands were visualized using the ChemiDoc System. Densitometry of the bands was performed by ImageLab 5.2 version software (Bio-Rad Laboratories, Hercules).

### Apoptosis assay

MCF-7 and MDA-MB-231 cells were treated with solvent DMSO/PBS (0.1%, vehicle control) or 2 µM CDDP, 2 µM CDDP + 0.1 µM or 5 µM ATV for 48 h. After treatment, cells were washed with PBS, resuspended with annexin V FITC-labeled antibodies and propidium iodide according to manufacturer’s instructions (Apoptosis Detection Kit II, BD Pharmingen, New Jersey, USA). BD Accuri ™ C6 flow cytometer, and CFlow^®^ software (BD AccuriTM, Franklin Lakes, NJ, USA) were used to quantify apoptotic and necrotic cells.

### Statistical analysis

All data were obtained from at least three independent experimental replicates and presented as mean ± SEM, unless otherwise indicated in the figure legends. Data analysis was performed using GraphPad Prism 9.0 (GraphPad Software). Differences between groups were determined using appropriate statistical tests noted in figure legends.

## Results

### Effect of CDDP treatment on MCF-7 and MDA-MB-231 cell proliferation and viability

**Fig. 1:**
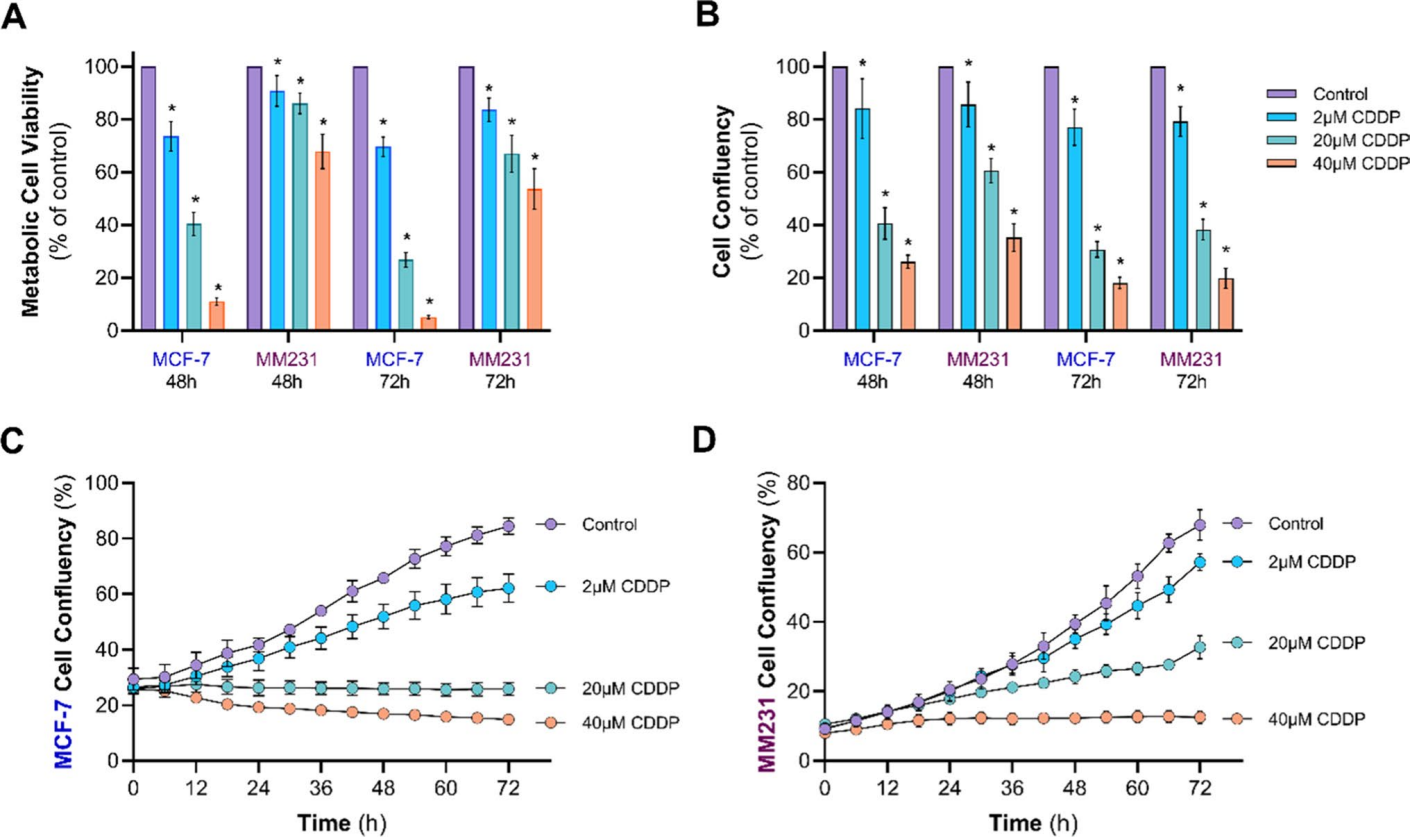
Effects of CDDP on MCF-7 and MDA-MB-231 cells. MCF-7 and MDA-MB-231 (MM231) cells were incubated in DMEM containing 10% FBS either with vehicle control (0.1% PBS) or CDDP (2, 20 or 40 µM) for 48 and 72 h. **A** Metabolic cell viability determined by MTT assay and **B** cell confluency by IncuCyte live cell imaging system. **A–B** Cell viability and confluency were presented as fold change to time-matched vehicle control. Data presented in (**B**) were obtained from the timeline data for **C** MCF-7, and **D** MDA-MB-231 (MM231) cells. Data are mean ± SEM of three independent experiments performed in quadruplicate (**A**) or with six technical replicates (**B–D**). *p < 0.05 vs. Control within each experiment by one-way ANOVA followed by Dunnett’s post hoc test (**A**&**B**)

We have previously reported that MCF-7 and MDA-MB-231 cells differ in their intracellular handling of fatty acids and in response to palmitate-induced apoptosis [22]. As such, we first set out to determine whether there are differences in the cytotoxicity of CDDP in MCF-7 and MDA-MB-231 cells. To achieve this, cells were incubated with CDDP (2, 20, 40 µM), and cell viability and cell growth after 48 and 72 h were determined. As expected, CDDP reduced MCF-7 and MDA-MB-231 cell viability (Fig. 1A) and confluency (Fig. 1B, C and D) in a dose-dependent manner. Cell-specific differences in the magnitude of the response to drug treatment in cell viability assays by MTT were observed, with MCF-7 cells being highly sensitive to increased CDDP concentrations (Fig. 1A). Yet, these differences between the two cell lines were not as striking when measuring cell growth based on cell confluency using the IncuCyte (Fig. 1B–D), suggesting that metabolic rearrangements in TNBC cells could be related to CDDP-induced cytotoxicity.

### ATV has cytotoxic activity in MCF-7 and MDA-MB-231 cell lines

**Fig. 2:**
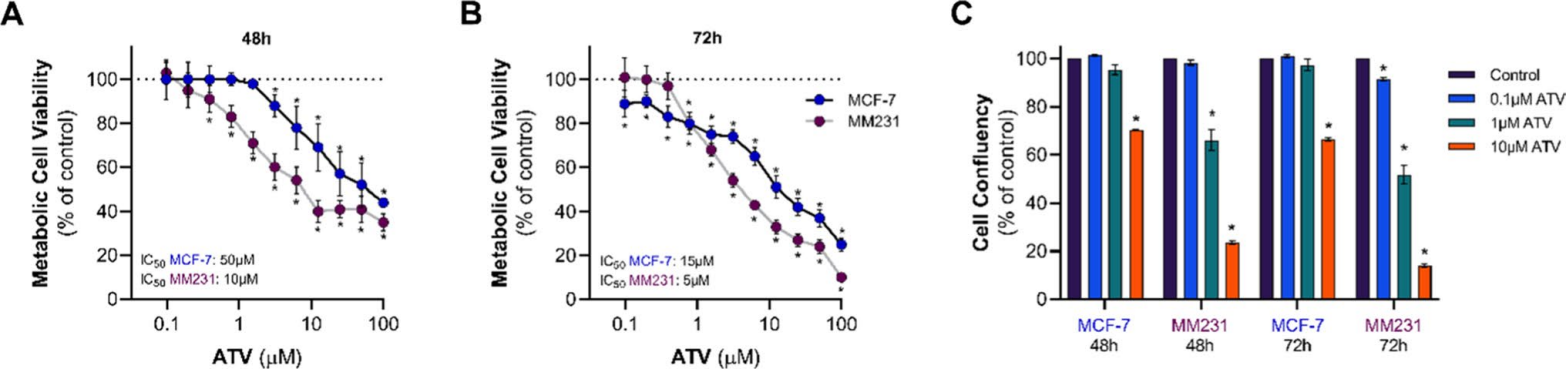
Cytotoxic effects of ATV in MCF-7 and MDA-MB-231 cells. ATV cytotoxicity on MCF-7 and MDA-MB-231 (MM231) cells cultured in DMEM containing 10% FBS, and IC_50_ values were calculated by MTT assay at **A** 48 h and **B** 72 h. **C** Effect of ATV treatment on cell confluency determined by IncuCyte and data presented as fold change to time-matched vehicle control. Data are expressed as mean ± SEM from three independent experiments performed in quadruplicate (**A**&**B**) or with six technical replicates (**C**). *p < 0.05 vs. Control (vehicle-treated cells) within each experiment by two-way ANOVA followed by Bonferroni post hoc test (**A**&**B**) or one-way ANOVA followed by Dunnett’s post hoc test (**C**). **A**&**B** IC_50_ values were calculated by nonlinear regression of log(inhibitor) vs. response using least squares as fitting method in a 4 parameters calculation with variable slope

Statins have been widely investigated as anti-cancer agents, and some studies suggest statins reduce BC-specific mortality significantly [23–25]. We therefore assessed the effectiveness of ATV to induce cytotoxicity in MCF-7 and MDA-MB-231 cells in MTT assays. MDA-MB-231 cells were most sensitive to the treatment with ATV compared to MCF-7 cells (Fig. 2A, B). Specifically, the IC_50_ cytotoxicity value of ATV in MCF-7 was 50 µM compared to 10 µM for MDA-MB-231 cells at 48 h (Fig. 2A), and 15 µM in MCF-7 cells and 5 µM for MDA-MB-231 cells at 72 h (Fig. 2B). The efficacy of ATV to reduce cell viability was similar in both cell lines following 48 h of drug treatment (approx. 60% vs. control), but not when cell lines were exposed to ATV for 72 h, where ATV efficacy was higher in MDA-MB-231 (approx. 90% vs. control) compared to MCF-7 cells (approx. 70% vs. control, Fig. 2B).

To complement these findings, a real-time IncuCyte assay was performed. MCF-7 and MDA-MB-231 cell lines were treated with ATV, and cell confluency was monitored for 72 h. In these experiments, MDA-MB-231 cell growth was affected in a dose- and time-dependent manner by ATV, and MDA-MB-231 cells were more sensitive to ATV when compared to MCF-7 cells (Fig. 2C). 10 µM ATV reduced MCF-7 viability by approximately 30% and by approximately 77% in MDA-MB-231 cells after 48 h (Fig. 2C). Interestingly, MCF-7 cell confluency remained unchanged at lower ATV concentrations even after 72 h of treatment. Moreover, there was no notable variation on MCF-7 cell confluency when comparing incubations at the highest ATV concentration (10 µM) between the 48 and 72 h data points. Taken together, these results suggested that ATV is more effective at suppressing MDA-MB-231 cell growth compared to MCF-7 cells.

### ATV enhanced the cytotoxic effect of CDDP in BC cells

Many statins have shown the potential to improve therapeutic outcomes of chemotherapy regimens in bladder cancer [26], osteosarcoma [27], liver [28], and pancreatic cancer [29]. As such, we evaluated the effect of ATV on CDDP sensitivity in BC cells. To achieve this, we performed experiments combining increasing concentrations of CDDP (0.064–40 µM) with 0.1 µM ATV, which is comparable to several pharmacokinetics studies conducted in hyperlipidemic patients (100–200 nM) [30] and still close to the estimated IC_50_ for ATV in MDA-MB-231 cells (5 µM) at 72 h.

**Fig. 3:**
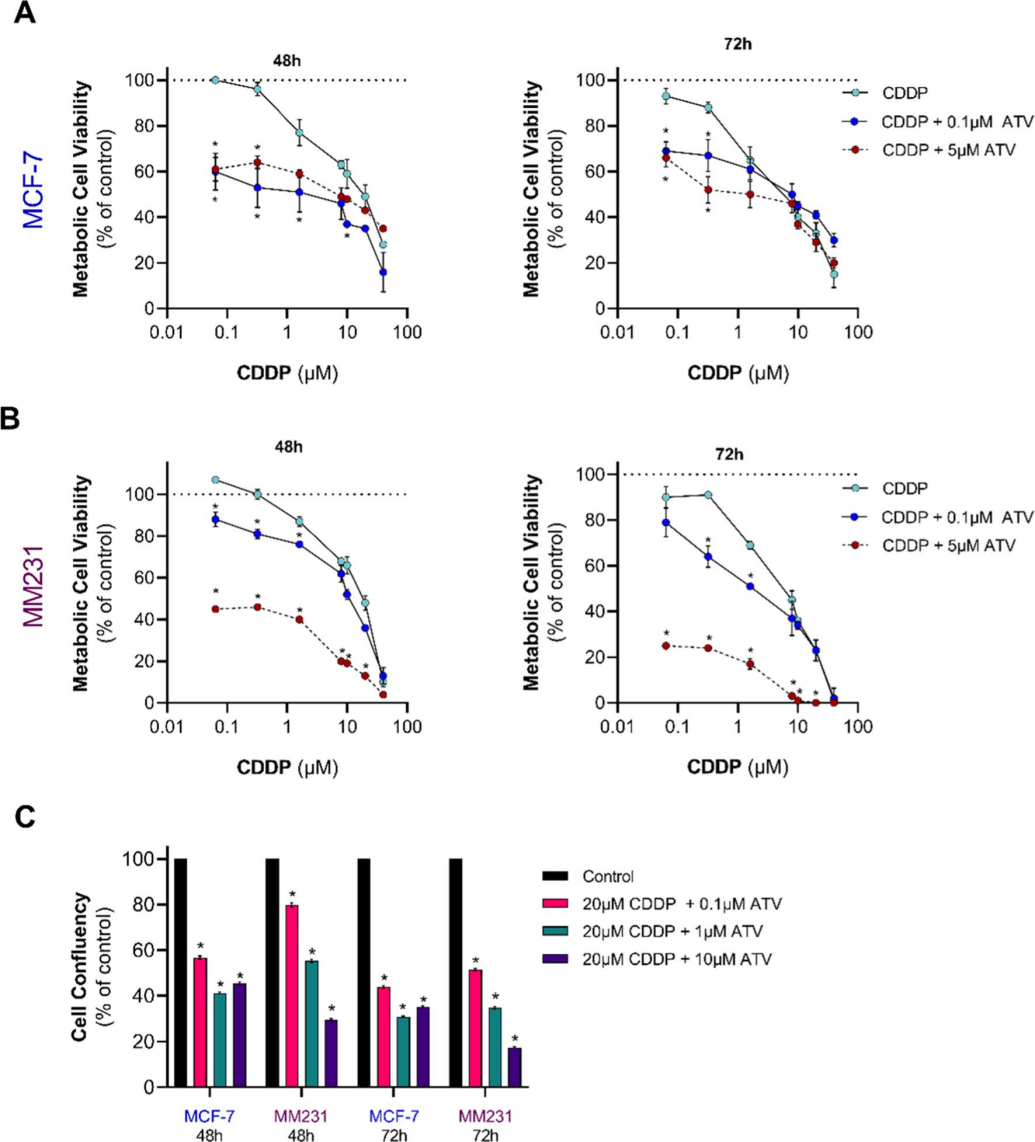
Cytotoxic effects of combined treatment with CDDP and ATV on MCF-7 and MDA-MB-231 cells. **A** MCF-7 and **B** MDA-MB-231 (MM231) cell viability in DMEM and 10% FBS media containing 0.64 to 40 µM of CDDP alone or in combination with ATV (0.1 µM or lower IC_50_ previously determined – 5µM) at 48 and 72 h. The data was expressed as a percentage of treated cells viability compared to untreated cells (Control). Control cell viability represented by horizontal dashed line indicating 100% cell viability. **C** MCF-7 and MDA-MB-231 cell confluency in response to solvent vehicle (Control) or co-administration of CDDP and ATV determined by IncuCyte at 48 and 72 h. Data are presented as fold change to time-matched vehicle control. Data are mean ± SEM of three independent experiments performed in quadruplicate (**A**–**B**) or with six technical replicates (**C**). *p < 0.05 vs. Control (vehicle-treated cells) within each experiment by two-way ANOVA followed by Bonferroni post hoc test (**A**&**B**) or one-way ANOVA followed by Dunnett’s post hoc test (**C**)

The combination of CDDP + ATV caused dose and time-dependent reductions in cell viability greater than CDDP alone (Fig. 3A&B). The estimated IC_50_ cytotoxicity was calculated (Table 1). Interestingly, MDA-MB-231 cells were more sensitive to the combined treatment of CDDP and ATV (Fig. 3B) compared to MCF-7 cells (Fig. 3A). In MDA-MB-231 cells, but not MCF-7 A, an additive cytotoxic effect was achieved when incubating cells with lower CDDP concentrations (0.064 µM – 10 µM) and with 5 µM ATV. This was reflected in the IC_50_ in MDA-MB-231 cells, as the combined treatment of CDDP and 5µM ATV was lower than the IC_50_ of CDDP by approximately 75- and 166-fold after 48 and 72 h, respectively (Table 1). Hence, co-treatment with ATV resulted in a robust increase of CDDP potency in MDA-MB-231 cells.

**Table 1:** IC_50_ values for CDDP either alone or in combination with ATV following 48 and 72 h exposure in MCF-7 and MDA-MB-231 cells

	MCF-7		MDA-MB-231	
Treatment	48 h	72 h	48 h	72 h
CDDP	15 µM	6.2 µM	15 µM	5 µM
CDDP + 0.1 µM ATV	4 µM	8 µM	10 µM	2.5 µM
CDDP + 5 µM ATV	9 µM	4 µM	0.2 µM	0.03 µM

The concentration–response inhibition equation with non-linear regression was used to calculate the IC_50_ from MTT assays

To further characterize the impact of CDDP effectiveness in the presence of ATV, we next compared cell viability using intermediate concentrations of ATV (0.1, 1, and 10 µM) and a constant concentration of CDDP (20 µM) (Fig. 3C). The intermediate concentration was chosen as MDA-MB-231 cell viability was completely abolished at higher concentrations of combinatorial CDDP and ATV treatment. In support of the data described above, proliferation of MCF-7 and MDA-MB-231 cells was decreased in the presence of CDDP and ATV (Fig. 3C). Of interest, MCF-7 cell proliferation was lowered by approximately 50% at all ATV concentrations analyzed. On the other hand, MDA-MB-231 cell proliferation was affected by the combined treatment in an ATV dose-dependent manner. At both 48 and 72 h time points, the growth inhibitory effect of 20 µM CDDP plus 10 µM ATV was greater in MDA-MB-231 cells (29%) than in MCF-7 cells (19%) (Fig. 3C). Although statins can promote apoptosis (32), the combined treatment of CDDP and ATV at these concentrations did not induce apoptosis compared to CDDP alone, as judged by annexin V FITC staining, in MCF-7 (Figure S1A) or MDA-MB-231 (Figure S1B) cells. Thus, the combined inhibitory effect of ATV and CDDP on cell proliferation suggests that cholesterol metabolism is a possible adaptive mechanism in BC cells to respond to CDDP treatment.

### ATV modulates CE levels in MCF-7 and MDA-MB-231 cells

**Fig. 4:**
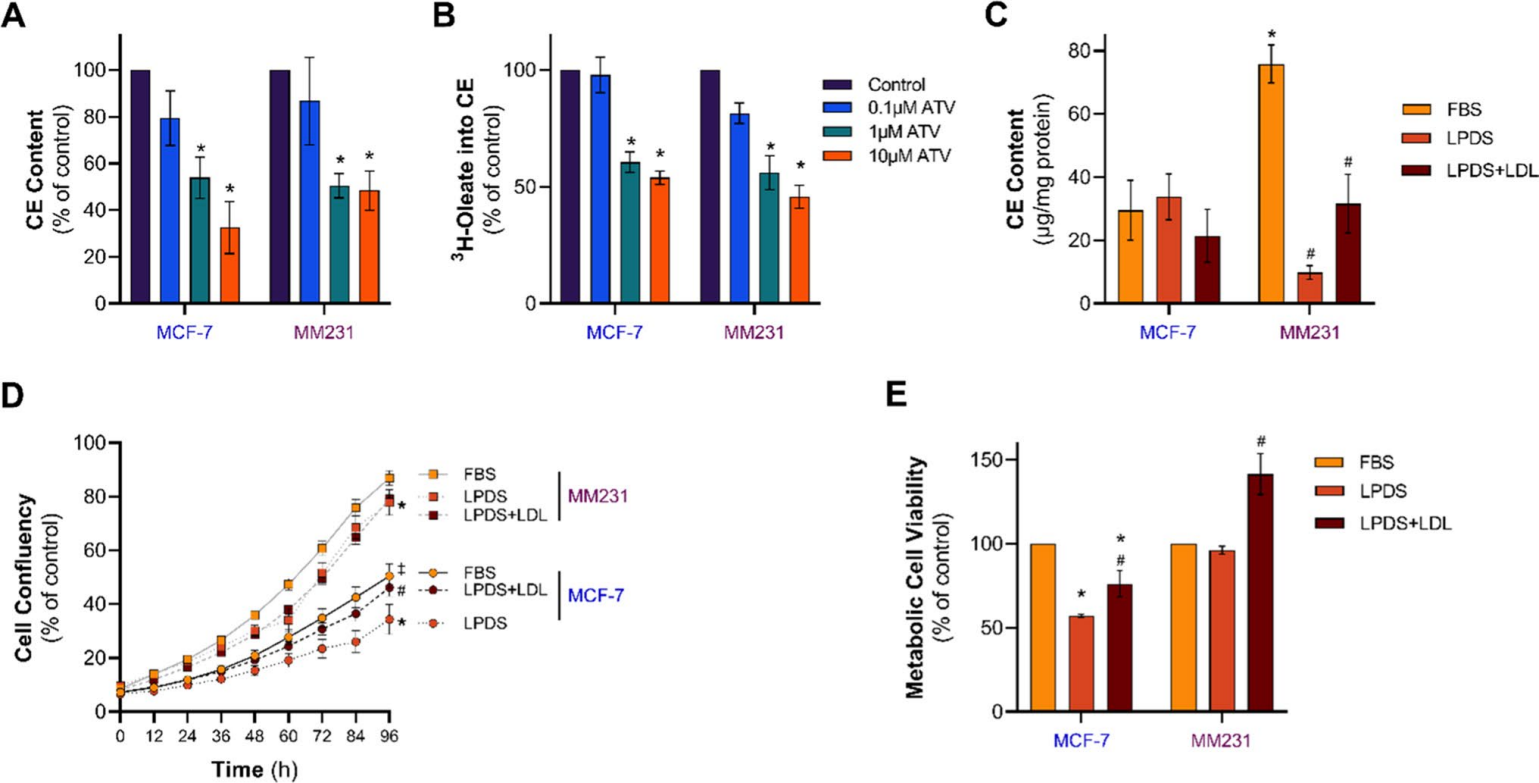
Cholesterol metabolism in MCF-7 and MDA-MB-231 cells in response to ATV treatment or cultured in modified media. MCF-7 and MDA-MB-231 (MM231) cell **A** cholesteryl ester (CE) content and **B** CE synthesis determined by ^3^ H-oleate incorporation into CE following culturing in DMEM containing 10% FBS either with vehicle control (0.1% DMSO) or ATV (0.1, 1 or 10 µM) for 48 h. MCF-7 and MM231 cell (**C**) CE content (**D**) proliferation by IncuCyte and (**E**) metabolic viability by MTT following culturing in DMEM containing 10% FBS, 10% LPDS or LPDS loaded with 50 µg/ml of human LDL. Data are mean ± SEM of at least three independent experiments performed in triplicate (**A**–**C**), quadruplicate (**E**) or with six technical replicates (**D**). *p < 0.05 vs. Control (vehicle-treated cells) within each experiment; #p < 0.05 vs. LPDS; ‡p < 0.05 vs. MM231 by one-way ANOVA followed by Dunnett’s post hoc test (A&B) or two-way ANOVA followed by Tukey’s post hoc test (**C**–**E**)

The differential response of MDA-MB-231 and MCF-7 cells to the combined treatment of CDDP and ATV suggested potential inherent differences in the sensitivity to ATV. While growing evidence demonstrates statins to provide anti-cancer activity via anti-proliferative and pro-apoptotic features, little is known whether reduced cholesterol availability upon statin treatment influences the development of chemoresistance. Cholesterol homeostasis is influenced by *de novo* cholesterol synthesis, uptake of extracellular cholesterol and CE turnover. To examine whether the effect of ATV on cell viability was due to reduced cholesterol synthesis and esterification, cells treated without or with 0.1–10 µM ATV were cultured in media supplemented with [^3^ H]-oleate. Indeed, CE content (Fig. 4A) and the rate of oleate incorporation into CE (Fig. 4B) were significantly decreased in both cell lines by approximately 50% after treatment with ATV compared to the control cells. These findings suggest that reduced CE content and CE synthesis in ATV-treated cells are likely to contribute to the increased efficacy of anti-cancer drugs.

To explore if differences in cholesterol homeostasis between the two cell lines would extend to alterations in the handling of LDL, the physiological and exogenous source of cholesterol, MCF-7 and MDA-MB-231 cells were cultured in medium supplemented with either FBS or lipoprotein-depleted serum (LPDS) with and without human LDL (50 mg/ml). CE levels were not altered in MCF-7 cells cultured in LPDS or in LPDS media supplemented with LDL (Fig. 4C). In contrast, CE levels were approximately four times higher in MDA-MB-231 cells compared to MCF-7 cells when cultured in FBS, reduced by 90% when cultured in LPDS-containing media, which was partially restored upon LDL supplementation (Fig. 4C). Supporting the non-radioactive determination of CE levels, the rate of oleate incorporation into CE was approximately four times higher in MDA-MB-231 cells compared to MCF-7 cells when cultured in FBS and reduced to 48% when cells were cultured in LPDS (Figure S2). These results suggest that MCF-7 cells exhibit compensatory mechanisms to overcome statin-induced cholesterol depletion or lipoprotein deprivation to maintain cellular CE levels. On the other hand, cellular cholesterol levels in MDA-MB-231 cells were highly responsive to a lipid-depleted environment and the availability of LDL-derived cholesterol, despite having higher intracellular CE stores when grown in normal conditions.

Cholesterol availability can influence cell proliferation [19, 31] and we next compared BC cell confluency grown in FBS, LPDS or LPDS together with LDL. The growth rate of MDA-MB-231 cells were higher than that of MCF-7 cells after 96 h (87% vs. 50%,) when cultivated in FBS (Fig. 4D). Interestingly, both MCF-7 and MDA-MB-231 cell proliferation kinetics were reduced when cultured in LPDS and compared to FBS. Yet, only MCF-7 cell proliferation was restored with LDL supplementation and was comparable to FBS-related growth kinetics (Fig. 4D). These results strongly suggest that the proliferation of MCF-7, but not MDA-MB-231, cells can be stimulated by the increased supply of exogenous and LDL-derived cholesterol. Consistent with these findings based on the IncuCyte, MCF-7 cell viability as determined by MTT assays was reduced by approximately 50% when cultured in LPDS compared to FCS, and partially recovered when LDL was added to LPDS-pretreated cells for an additional 24 h (76%; Fig. 4E). Interestingly, MDA-MB-231 cell viability was comparable in FBS and LPDS-containing media but was increased by 50% when LPDS was supplemented with LDL (Fig. 4E), indicating that an LDL-rich environment can stimulate BC viability irrespective of the BC subtype.

### CDDP sensitivity is associated with changes in expression patterns of key players in cholesterol homeostasis

**Fig. 5:**
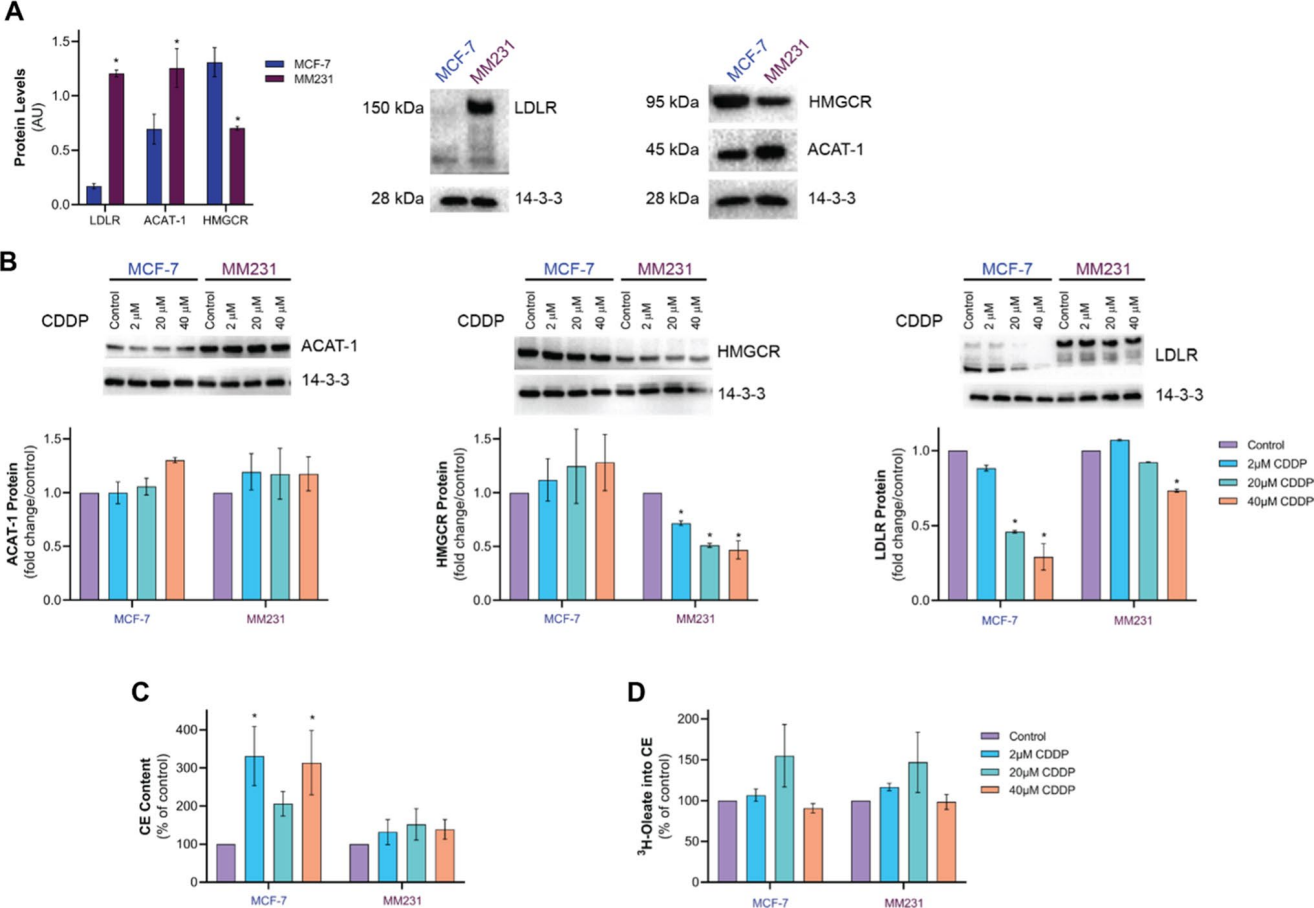
Effect of CDDP on CE metabolism in MCF-7 and MDA-MB-231 cells. **A** Representative immunoblots and densitometric quantitation of LDLR, ACAT-1, and HMGCR protein levels in MCF-7 and MDA-MB-231 (MM231) cells. **B** Representative immunoblots and densitometric quantitation of ACAT-1, HMGCR and LDLR protein levels in MCF-7 and MDA-MB-231 cells treated with the solvent vehicle (Control) or CDDP for 48 h. MCF-7 and MDA-MB-231 cell (**C**) CE content and (**D**) cholesterol esterification following culturing in solvent vehicle (Control) or CDDP for 24 h. Data are mean ± SEM of at least three independent experiments performed in triplicate. *p < 0.05 vs. MCF-7 cells by unpaired Student’s t-test (A). *p < 0.05 vs. Control (vehicle-treated cells) within each experiment by one-way ANOVA followed by Dunnett’s post hoc test (**B**–**D**)

Cytotoxic chemotherapy can trigger metabolic adaptations in human tumors, and often include changes in lipid homeostasis [13], such as the upregulation of cholesterol synthesis, uptake and esterification [32]. These metabolic features are often accompanied by CE accumulation, all of which support cancer proliferation and survival [19, 33]. Given the ability of ATV to improve CDDP efficacy in MDA-MB-231 cells (Fig. 3), we next measured aspects of CE metabolism in BC cells after acute CDDP exposure. We hypothesized that differences in the expression levels of key players in cholesterol synthesis (HMGCR), cholesterol uptake (LDLR) and cholesterol esterification (ACAT-1), leading to changes in lipid metabolism, could in part explain the disparate cytotoxic effect of CDDP on BC cell proliferation and viability. Therefore, cell lysates from MDA-MB-231 and MCF-7 cells were prepared and analyzed by western blotting (Fig. 5). In the absence of CDDP, the protein levels of HMGCR were 1.8-fold higher in MCF-7 cells compared to MDA-MB-231 cells, whereas LDLR (7.5-fold) and ACAT-1 (1.6-fold) were elevated in MDA-MB-231 cells compared to MCF-7 cells (Fig. 5A). CDDP treatment did not alter ACAT-1 protein levels in MCF-7 or MDA-MB-231 cells (Fig. 5B), whereas HMGCR protein levels were reduced by 28% and 52% in MDA-MB-231 cells treated with 2 µM and 20 µM CDDP, respectively, but remained unaltered in MCF-7 cells (Fig. 5B). Interestingly, LDLR protein levels were reduced in MCF-7 cells to 45% and 29% of control levels in response to 20 µM and 40 µM CDDP, respectively; however, LDLR was decreased by 27% in MDA-MB-231 cells only at the highest concentration (Fig. 5B). Besides these CDDP-induced changes in the expression of key proteins in cholesterol homeostasis, CDDP treatment significantly increased CE levels in MCF-7 cells, and a trend for increased levels of CE was also observed in MDA-MB-231 cells (Fig. 5C). These findings correlated with a trend towards an increased rate of CE synthesis at low CDDP concentrations (2–20 mM) that was not apparent at higher CDDP concentrations in both BC lines (Fig. 5D).

### ATV impacts CDDP sensitivity in MDA-MB-231 cells by regulating key enzymes involved in CE metabolism

**Fig. 6:**
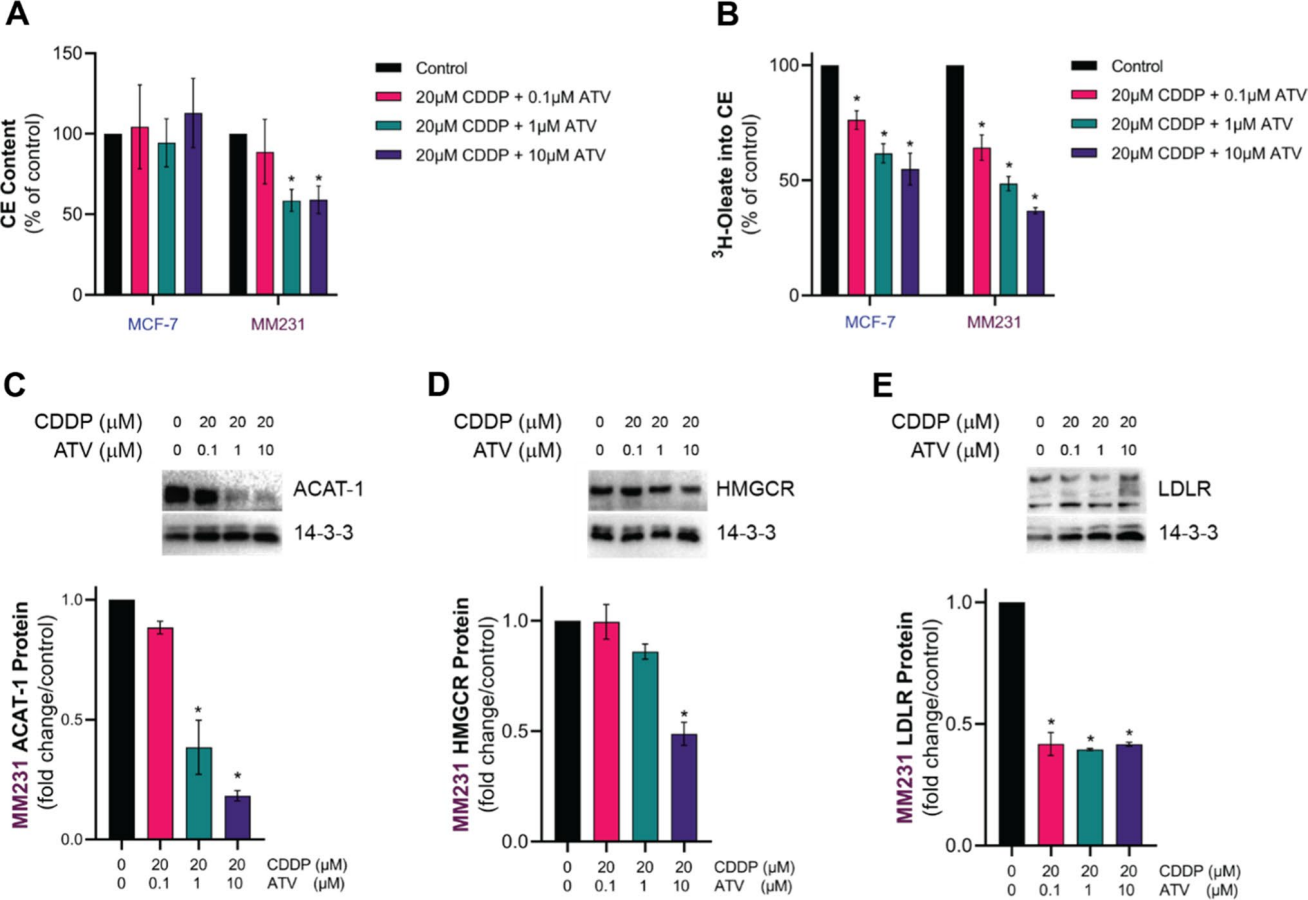
BC cell CE metabolism after combined treatment with CDDP and ATV. MCF-7 and MDA-MB-231 (MM231) cell **A** CE content and **B** cholesterol esterification following culturing in growth media (Control) or media containing CDDP (20 µM) plus ATV for 48 h. Representative immunoblots and densitometric quantitation of **C** ACAT-1, **D** HMGCR and **E** LDLR protein levels in MDA-MB-231 cells treated with the solvent vehicle (Control) or CDDP plus ATV. Data are mean ± SEM of at least three independent experiments performed in triplicates. *p < 0.05 vs. Control (vehicle-treated cells) within each experiment by one-way ANOVA followed by Dunnett’s post hoc test.

We next investigated the effect of CDDP and ATV co-treatment on CE metabolism in MCF-7 and MDA-MB-231 cells. There was no change in the CE levels of MCF-7 cells in response to 20 µM CDDP when combined with increasing concentrations of ATV (0.1–10 µM; Fig. 6A), whereas CE levels in MDA-MB-231 cells were reduced to approximately 60% of control (Fig. 6A). This reduction in CE levels in MDA-MB-231 cells was associated with a reduction in the cholesterol esterification rate (Fig. 6B), suggesting that the reduced supply of endogenous cholesterol due to ATV exposure, but not CDDP, was responsible for this observation. Interestingly, despite the reduction in cholesterol esterification rate in MCF-7 cells co-treated with CDDP and ATV, this did not reduce total CE levels (Fig. 6A). These observations suggest that MCF-7 and MDA-MB-231 cells store and metabolize esterified cholesterol in the presence of CDDP and ATV differently, which could contribute to the differential anti-cancer drug sensitivity observed above (Fig. 3).

Due to the more prominent effects of CDDP and ATV in MDA-MB-231 than in MCF-7 cells, we next measured the protein levels of LDLR, ACAT-1, and HMGCR in MDA-MB-231 cells exposed to CDDP and ATV. In agreement with the previous findings that showed decreased cholesterol esterification in MDA-MB-231 cells (Fig. 4B), ACAT-1 protein levels were reduced by 70% following treatment with 20 µM CDDP and 1 µM or 10 µM ATV concomitantly for 24 h (Fig. 6C). The combination of CDDP and ATV also downregulated the expression of HMGCR (Fig. 6D) and LDLR (Fig. 6E) by 50% in MDA-MB-231 cells. As CDDP acute treatment alone or single treatment with ATV did not modulate the expression of ACAT-1 (Figure S3), one might postulate an additive effect of CDDP and ATV in MDA-MB-231 cells that leads to a major deregulation of cholesterol homeostasis, compromising cholesterol uptake, synthesis, and esterification. Combined, these changes will ultimately lead to CE depletion and cholesterol availability, resulting in increased cell death since cholesterol is a key prerequisite to sustain the metabolic requirements that accompanies BC progression [34].

### ATV re-sensitizes MDACR cells to CDDP

**Fig. 7:**
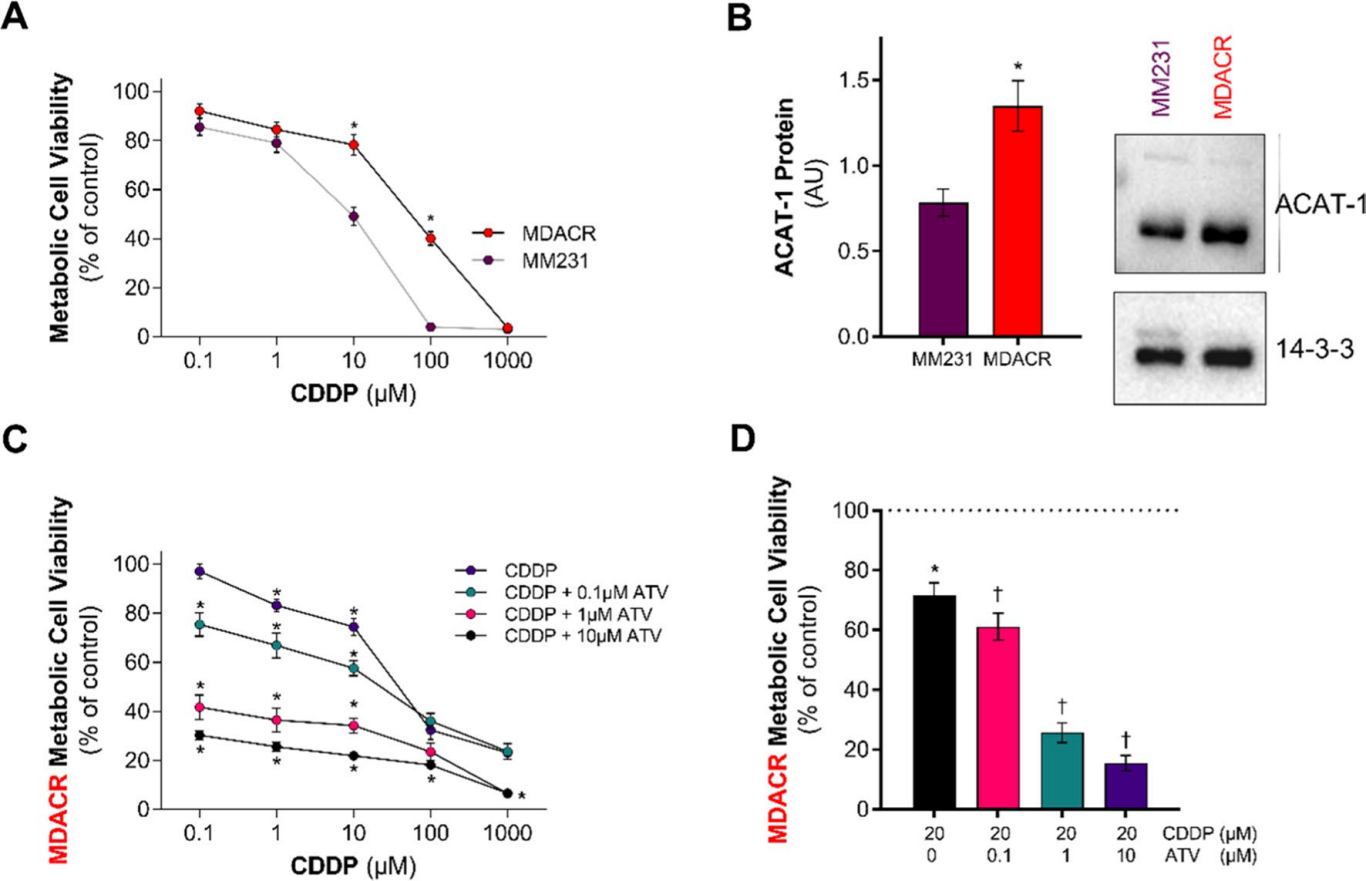
Effect of combined treatment with CDDP plus ATV on MDACR cells. **A** MDACR and MDA-MB-231 (MM231) metabolic cell viability determined by MTT following culturing in media containing CDDP for 48 h. **B** Representative immunoblots and densitometric quantitation of ACAT-1 in MDA-MB-231 and MDACR cells. (**C**) MDACR cell viability following culturing media containing CDDP alone (ranging from 0.1µM to 1mM) or with ATV (0.1, 1 or 10 µM) for 48 h. Data were normalized to 0.1 µM CDDP only group. (D) MDACR cell viability following culturing in media containing 20 µM CDDP alone or in combination with ATV (0.1, 1 or 10 µM) for 48 h. The data was expressed as percentage of treated cells viability compared to untreated cells (Control). Control cell viability represented by horizontal dashed line indicating 100% cell viability. Data are mean ± SEM at least three independent experiments performed in triplicate (**B**) or quadruplicate (**A**, **C**&**D**). *p < 0.05 vs. MDA-MB-231 cells by two-way ANOVA followed by Šídák’s post hoc test (**A**) or by unpaired Student’s t-test (B). *p < 0.05 vs. 0.1 µM CDDP only cells by one-way ANOVA followed by Tukey’s post hoc test (**C**). *p < 0.05 vs. untreated cells (Control), †p < 0.05 vs. 20 µM CDDP treated cells by one-way ANOVA followed by Tukey’s post hoc test (**D**)

The outcome of the experiments described above shows that a combination of CDDP and AVT modulated MCF-7 and MDA-MB-231 cell cholesterol metabolism and cell viability in the acute setting. Next, we determined whether changes in cholesterol homeostasis could represent long-term adaptive mechanisms to CDDP treatment. To test this hypothesis and consider the clinical challenge of treatment resistance, we investigated the effect of the combined treatment in an in-house generated CDDP-resistant cell line (MDACR) derived from the parental MDA-MB-231 lineage. The MDACR cell line exhibited a 5 to 12-fold increased resistance to CDDP and an IC_50_ seven times higher (88.2 µM) than its parental cell line (12.6 µM) (Fig. 7A). Upon CDDP treatment, MDACR cells displayed a fibroblast-like phenotype (Figure S4) and, strikingly, cross-resistance to other chemotherapeutic drugs (Figure S5) but not to ATV (Figure S6). These changes correlated with a remarkable increase in ACAT-1 protein levels in the MDACR cell line (2.5-fold) compared to the parental MDA-MB-231 cells (Fig. 7B). As such, we tested whether ATV treatment could modulate MDACR cell viability and its CDDP-resistant phenotype. In fact, ATV co-treatment re-sensitized MDACR cells to CDDP in a dose-dependent fashion (Fig. 7C and D). Hence, administering cholesterol-lowering statins might be a powerful approach to overcoming acquired CDDP resistance in TNBC cell lines.

## Discussion

Treatment resistance is a major clinical challenge in BC management and contributes to poor patient outcomes [35]. The results of this study emphasize the important role that cholesterol metabolism plays in BC progression as an adaptive mechanism in response to CDDP treatment. We showed that CDDP alters cholesterol metabolism in luminal A (MCF-7) and TNBC (MDA-MB-231) cell models, and that CDDP together with ATV reduced cell proliferation and cell viability to a greater extent than CDDP alone, especially in MDA-MB-231 cells. Furthermore, the combination of CDDP and ATV dramatically altered cellular cholesterol metabolism in these cells. These findings indicate that the elevated susceptibility of MDA-MB-231 cells to co-administration of CDDP and ATV, compared to MCF-7 cells, was associated with an increased reliance on CE availability. Moreover, ATV restored CDDP sensitivity in CDDP-resistant MDA-MB-231 cells. Collectively, our data implicate the upregulation of CE storage as an adaptive response that contributes to chemotherapy resistance.

A recently reported case study showed CDDP reduced metastatic legions in heavily pre-treated TNBC when used as monotherapy [36]. Despite the initial successful response to platinum-based chemotherapy in many settings, including TNBC, the acquirement of resistance is associated with many gene expression changes [37], ultimately resulting in disease relapses. Here, we assessed the role of cholesterol metabolism in the acute and chronic settings using cell culture. Interestingly, we observed that MDA-MB-231 cells were intrinsically more resistant to CDDP than MCF-7 cells. This was the first hint that CDDP possibly acted differentially on the cellular metabolism of TNBC cells compared to luminal A BC cells. This differential response to CDDP treatment may be due to many factors alongside differences in cholesterol metabolism; fully defining the underlying differences in CDDP sensitivity was beyond the scope of our study. Targeting cholesterol metabolism in BC treatment continues to be explored in many ongoing clinical trials, with over 24 trials currently testing the effects of statins in BC (clinicaltrials.gov). Of these, notably, two are evaluating the benefits of ATV in association with conventional chemotherapy in TNBC patients (NCT03358017; NCT03872388). Nonetheless, whether cholesterol metabolism contributes to the development of chemoresistance to CDDP in TNBC cells remains to be fully defined.

In recent decades, many studies have shown that chemotherapeutic drugs can lead to metabolic disorders in patients, including changes in serum lipids and lipoproteins [32, 38]. For example, mice treated repeatedly with paclitaxel and CDDP had increased circulating cholesterol levels [39]. Similarly, CDDP-resistant ovarian cancer cells exhibit increased expression of key cholesterol metabolism genes, compared to treatment sensitive cells, including sterol regulatory-element binding protein 2, LDLR, and HMGCR, thereby suggesting that upregulated cholesterol metabolism might contribute to CDDP resistance in ovarian cancer [40]. While acute CDDP exposure decreased LDLR protein levels in both BC cell lines studied in the present work, a reduction in HMGCR levels was observed only in MDA-MB-231 cells, suggesting the occurrence of cancer cell type-specific changes in cholesterol handling and CE synthesis. Given a trend of increased ACAT-1 expression, concomitantly with higher CE content after CDDP treatment, this enzyme plays an important role in triggering metabolic changes in BC cells, contributing to the increased proliferation rate of MDA-MB-231 and different responses to anti-cancer therapy. In fact, elevated CE storage mediated through upregulated ACAT-1 activity may provide growing cells with immediately available building blocks for membrane construction and reduce the need for *de novo* lipid synthesis [41].

Cancer cells display metabolic reprogramming to better utilize lipids for tumor development and progression [4], and targeting these pathways with a combination of low-toxic compounds may become an alternative therapeutic intervention. Emerging evidence suggests that a decrease of CE levels via ACAT-1 inhibition significantly improves chemotherapy in melanoma [42] and in prostate cancer cell growth [19, 43]. Moreover, reducing fatty acid metabolism by inhibiting fatty acid synthase (FASN), enhanced the response to CDDP and sensitized resistant ovarian and mammary cells [44, 45]. In BC models, FASN inhibition improved doxorubicin, docetaxel, paclitaxel, and vinorelbine chemotherapy [46, 47]. In this study, the reduction of cholesterol esterification in MDA-MB-231 cells following exposure to combined treatment with CDDP and ATV resulted in inhibiting all key proteins involved in CE formation (Fig. 6), highlighting the potential significance of improving CDDP response with ATV co-treatment in TNBC.

Multiple mechanisms have been associated with the acquisition of a chemoresistant phenotype by cancer cells, including drug inactivation, drug efflux, apoptosis suppression, increased DNA repair [36]. However, the role of CE metabolism in CDDP resistance in BC cells remains unclear. Results presented here suggest that not only *de novo* cholesterol synthesis but also exogenous cholesterol, delivered through the uptake of LDL, can contribute to increased CE storage and, consequently, resistance to CDDP. Hence, this correlates with elevated LDLR accelerating TNBC tumor growth in hyperlipidemic mouse models [7], and elevated plasma cholesterol levels contributing to drug resistance [48]. Notably, BC patients with obesity had worse clinical outcomes in response to paclitaxel and carboplatin treatment than non-obese patients [49]. This suggests that the dyslipidemia often associated with obesity, including increased circulating cholesterol and CE, can play a critical role in the tumor microenvironment, influencing cell behavior and response to CDDP.

We have previously reported that MCF-7 and MDA-MB-231 cells differ in their intracellular handling of fatty acids and in response to palmitate-induced apoptosis [22]. Here, we extend these observations and report that these cells also differ in the levels of ACAT-1, LDLR, and HMGCR (Fig. 5A). Moreover, BC cells have distinct CE etiology so that most of the CE supply in TNBC models like MDA-MB-231 requires elevated ACAT-1 activity and overexpression of LDLR while, in MCF-7, cholesterol and CEs are mostly provided by the cholesterol synthesis route, benefiting only from elevated HMGCR activity. Cholesterol homeostasis is fine-tuned and regulated by feedback mechanisms that allow elevated free cholesterol levels to activate ACAT-1 to catalyze its esterification and storage, while simultaneously inhibiting HMGCR [50]. Indeed, free cholesterol levels were substantially higher in MDA-MB-231 compared to MCF-7 cells (Figure S7). Also, our findings suggest a shift towards cholesterol production by TNBC cells under cholesterol deprivation, allowing them to recover efficiently from this condition. We speculate that elevated ACAT-1 levels may allow MDA-MB-231 cells to recover more rapidly from stress, such as drug treatment or cholesterol deprivation. As TNBC cells express elevated ACAT-1 levels, which is coupled to a higher growth rate, we speculate that increased amounts of CE generated by ACAT-1 and stored in lipid droplets are then available for hydrolysis to produce cholesterol, reducing the pool of intracellular CE, and sustaining cell proliferation.

The regulation of cholesterol homeostasis in the various BC subtypes and its contribution to resistance against therapy remains to be fully understood. Previous studies conducted in tumor tissue samples reported a positive correlation between CE content and clinicopathological parameters, such as the occurrence of high-grade tumors [51]. Indeed, TNBC specimens expressed higher levels of LDLR and ACAT-1 and displayed elevated intratumoral CE levels compared to luminal A BC [51]. However, in these studies, HMGCR mRNA levels were comparable between the BC subtypes. Furthermore, previous microarray studies also demonstrated that basal-like BC exhibit elevated ACAT-1 levels [52, 53], thus corroborating our results. Nonetheless, our data points to the fact that ACAT-1 expression is greater in MDA-MB-231 cells chronically exposed to CDDP (MDACR), which might reflect events that occur in response to anti-cancer treatments. Remarkably, as shown here, ATV re-sensitized MDACR cells to CDDP. Fromigué et al. [27] suggested that high doses of ATV increased drug sensitivity through the modulation of matrix metalloprotease 2 in osteosarcoma cells. On the other hand, Guo and collaborators [28] showed that liver cancer cells (Huh-7) became more sensitive to CDDP in the presence of 100 µM ATV. Here, we report a correlation between the modulation of ACAT-1 expression upon exposure to CDDP in combination with ATV in MDA-MB-231 cells. Moreover, we demonstrate the importance of ACAT-1 and cholesterol availability in acquiring chemoresistance to CDDP in a TNBC cell model. Considering that drastic downregulation of ACAT-1 correlated with the enhanced anti-tumor activity of CDDP in combination with ATV in MDA-MB-231, one can speculate that restoration of CDDP activity observed in the presence of ATV in CDDP-resistant TNBC also results from ACAT-1 downregulation and reduced CE availability.

While our study provides new insights into the links between cholesterol metabolism, CDDP and BC biology in cell culture experiments, there are some limitations. The scope of our study was limited to investigating the mechanism of chemoresistance in human TNBC and Luminal A cells, hypothesizing that changes in cholesterol metabolism are early events that can support acquired treatment resistance. Therefore, we performed experiments in commonly used immortalized tumorigenic cell lines, MDA-MB-231 and MCF-7. It should be noted that the scope of our study did not include examining whether our observations were exclusive to models of BC. Future studies should examine CDDP and statins’ effect in a range of BC cells. Clinically, our question focused on whether there is a synergistic benefit to patients that will be treated with CDDP and thereby improve patient outcomes. While our cell culture-based studies provide a platform, future studies should aim to establish evidence in pre-clinical models before widespread deployment in the clinic. However, as noted above, ongoing clinical trials are evaluating the efficacy of including statins alongside conventional anti-cancer therapies. In any event, it is conceivable that ATV and other statins will have significant effects on the liver and, thereby extra-tumor levels of cholesterol to influence disease progression, which we cannot explore in vitro. However, it should be noted that current pre-clinical models of acquired treatment resistance are suboptimal, if they exist at all.

## Conclusion

Overall, this study suggests that ATV increased the sensitivity of CDDP-resistant MDA-MB-231 cells through reduced ACAT-1 activity and changes in cholesterol homeostasis. This regulatory circuit could be utilized as a chemosensitizer for CDDP-resistant BC cells and further supports concepts targeting cholesterol metabolism, which is safe and cost-effective, as adjuvant therapy for platinum-based chemotherapeutics in BC.

## Supplementary information

**Supplementary material 1: Figure S1 **Effect of combined CDDP and ATV treatment on apoptotic rates in MCF-7 and MDA-MB-231 cell lines. **Figure S2 **Effect of cholesterol deprivation on cholesterol esterification.  **Figure S3 **Effect of ATV on ACAT-1 expression. **Figure S4 **Breast cancer cell morphology. **Figure S5** Analysis of cross-resistance to paclitaxel and doxorubicin in MDA-MB-231 and MDACR cells. **Figure S6** Comparison of ATV sensitivity in MDA-MB-231 and MDACR cells. **Figure S7** Intracellular free cholesterol content in MCF-7 and MDA-MB-231 cells. **Figure S8** Immunoblots used as representative blots assembled in various figures of the manuscript

## Data Availability

The authors confirm that the data supporting the findings of this study are available within the article and its supplementary material.

## Abbreviations

ACAT-1: Acyl-Coenzyme A:cholesterol acyltransferase 1
ATV: Atorvastatin
BC: Breast cancer
CE: Cholesteryl ester
CDDP: Cisplatin
FBS: Fetal bovine serum
HMGCR: 3-Hydroxy-3-methylglutaryl-CoA reductase
LDL: Low-density lipoprotein
LDLR: Low-density lipoprotein receptor
LPDS: Lipoprotein-deplete serum
MM231: MDA-MB-231 cells
MTT: Thiazolyl blue tetrazolium blue
TNBC: Triple negative breast cancer.
