# Supplementary material for: Atorvastatin improves cisplatin sensitivity through modulation of cholesteryl ester homeostasis in breast cancer cells
Source: Discov Oncol. 2022 Dec 8;13:135. doi: 10.1007/s12672-022-00598-8 (PMC9732177; doi:10.1007/s12672-022-00598-8)
Supplement: Supplementary file 1 — Supplementary material 1: Figure S1 Effect of combined CDDP and ATV treatment on apoptotic rates in MCF-7 and MDA-MB-231 cell lines. Figure S2 Effect of cholesterol deprivation on cholesterol esterification. Figure S3 Effect of ATV on ACAT-1 expression. Figure S4 Breast cancer cell morphology. Figure S5 Analysis of cross-resistance to paclitaxel and doxorubicin in MDA-MB-231 and MDACR cells. Figure S6 Comparison of ATV sensitivity in MDA-MB-231 and MDACR cells. Figure S7 Intracellular free cholesterol content in MCF-7 and MDA-MB-231 cells. Figure S8 Immunoblots used as representative blots assembled in various figures of the manuscript [file 12672_2022_598_MOESM1_ESM.pdf]

Atorvastatin Improves Cisplatin Sensitivity Through Modulation of Cholesteryl Ester Homeostasis in Breast Cancer Cells

Diandra Zipinotti dos Santos[1], Isabella dos Santos Guimaraes[2], Mariam F. Hakeem-Sanni[3], Blake J. Cochran[4], Kerry-Anne Rye[4], Thomas Grewal[5], Andrew J. Hoy [3#], Leticia B.A. Rangel[1,6,7#]

[1] Biotechnology Program/RENORBIO, Health Sciences Center, Universidade Federal do Espírito Santo, Vitoria, ES, Brazil

[2] Division of Clinical Research, Research Center, Instituto Nacional do Câncer, Rio de Janeiro, Brazil

[3] School of Medical Sciences, Charles Perkins Centre, Faculty of Medicine and Health, The University of Sydney, Sydney, New South Wales, Australia.

[4] School of Medical Sciences, Faculty of Medicine, UNSW, Sydney, New South Wales, Australia

[5] School of Pharmacy, Faculty of Medicine and Health, The University of Sydney, Sydney, New South Wales, Australia

[6] Biochemistry Program, Health Sciences Center, Universidade Federal do Espirito Santo, Vitoria, ES, Brazil

[7]Department of Pharmaceutical Sciences, Universidade Federal do Espírito Santo, Vitória, Brazil

Supplementary Data

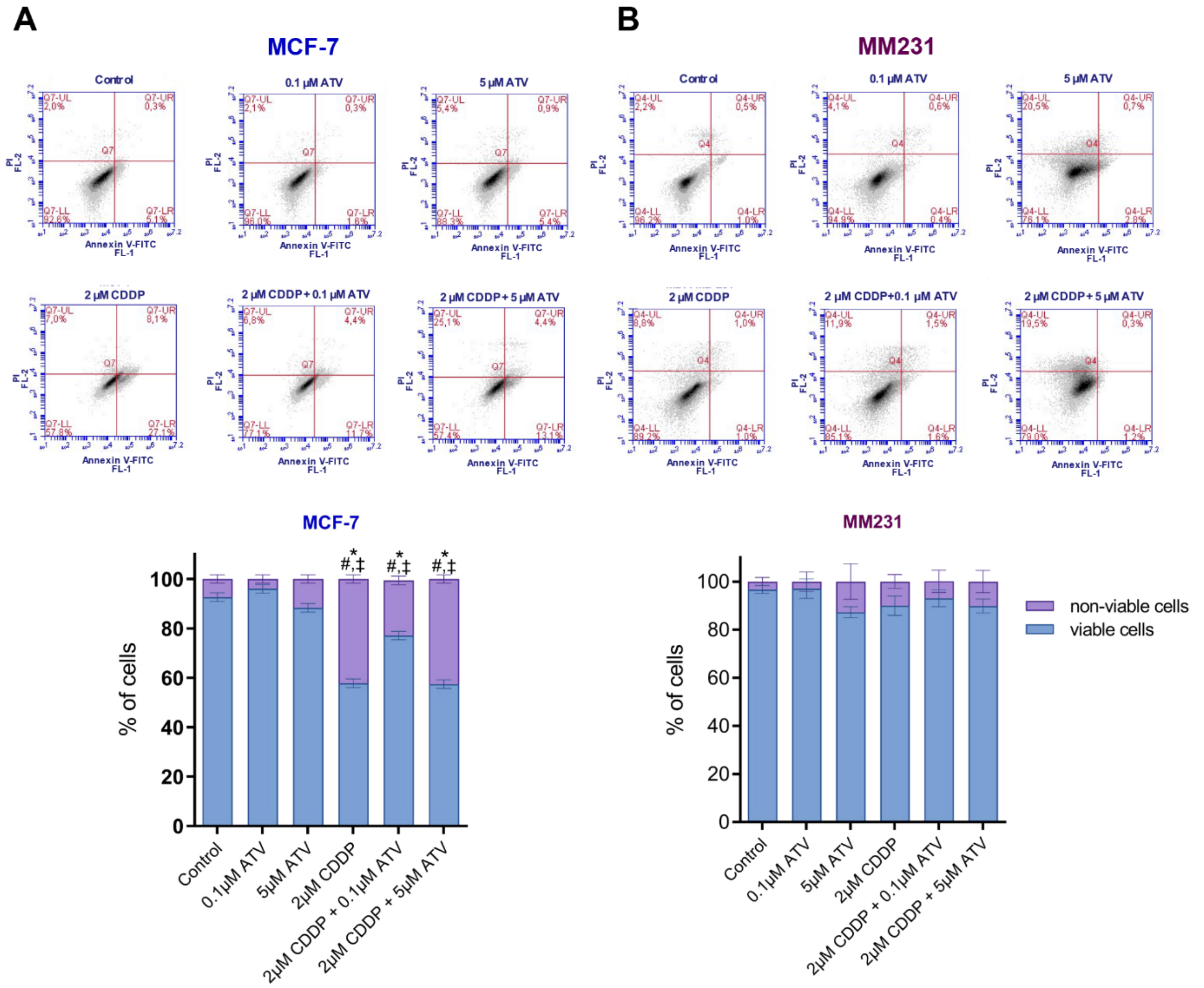

Figure S1: Effect of combined CDDP and ATV treatment on apoptotic rates in MCF-7 and MDA-MB-231 cell lines.

Induction of apoptosis by CDDP and ATV in BC cells as determined by PI/annexin V staining assay. (A) MCF-7 and (B) MDA-MB-231 (MM231) cells were treated vehicle solvent (control), 0.1 µM ATV, 0.1 µM (IC_{50}) ATV (top panels), or 2 µM CDDP alone, 2 µM CDDP + 0.1 µM ATV or 2 µM CDDP + 5 µM ATV (bottom panels). Flow cytometric analysis and quantification of results are shown after treatment for 48 h. Data are represented as mean ± SEM of three independent experiments performed in triplicate. *p <0.05 vs Control (vehicle treated cells), #p<0.05 vs 0.1 µM ATV; ‡p<0.05 vs 5 µM ATV by one-way ANOVA followed by Tukey's post hoc test (C&D).

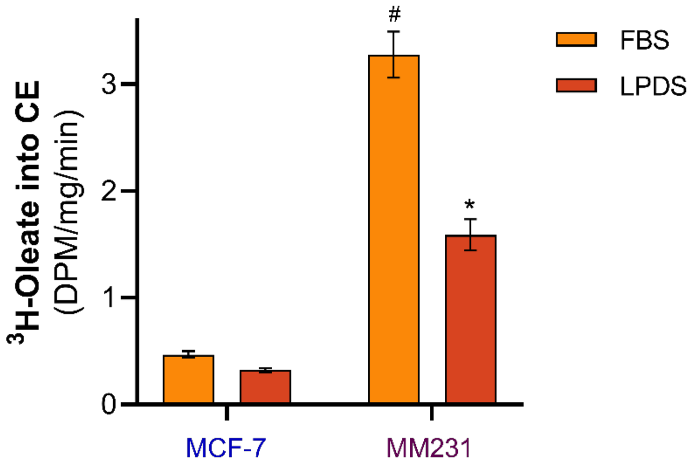

Figure S2: Effect of cholesterol deprivation on cholesterol esterification.
Incorporation of radiolabeled oleate into CE in MCF-7 and MDA-MB-231 (MM231) cells. BC cells were plated and cultivated DMEM plus 10% FBS or DMEM plus 10% LPDS for 48h. Radiolabeled oleate was added and incorporation into CE was determined. Data are represented as mean ± SEM of three independent experiments, each performed in triplicate, *p <0.05 vs LPDS, #p <0.05 vs MCF-7 FBS by two-way ANOVA followed by Tukey's post hoc test.

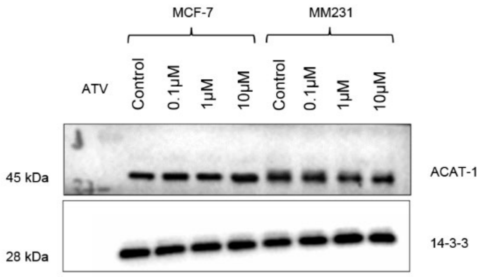

Figure S3: Effect of ATV on ACAT-1 expression.
Representative immunoblots of three independent measures of ACAT-1 protein levels in MCF-7 and MDA-MB-231 (MM231) cells cultured in media containing ATV for 48h.

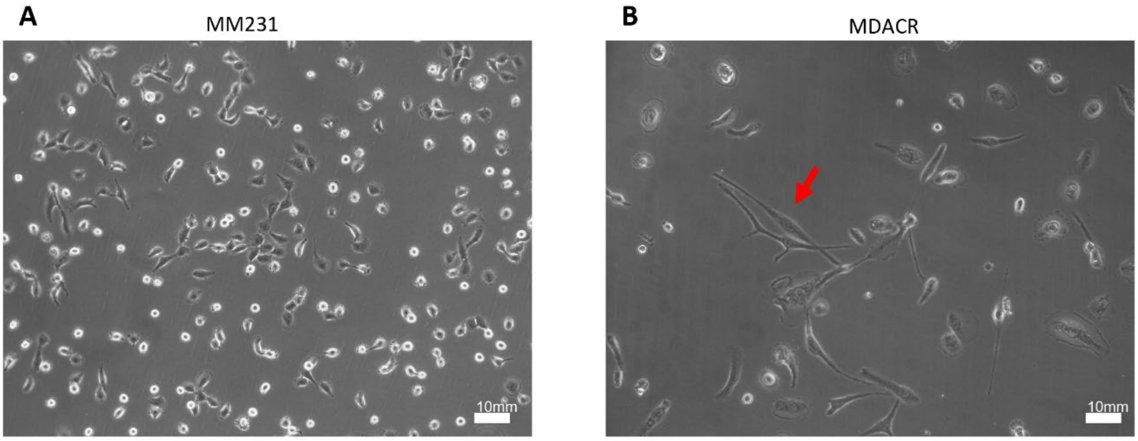

Figure S4: Breast cancer cell morphology.
Brightfield images of (A) MDA-MB-231 (MM231) and (B) MDACR cells. MDACR cells have distinct morphological changes exposed to CDDP (indicated by the red arrow). Cell morphology was analyzed in a Zeiss Observer Z1 microscope, and all images were acquired by Axio-Vision Rel. 4.8 software (Carl Zeiss). Magnification 10x. The scale bars are 10 mm.

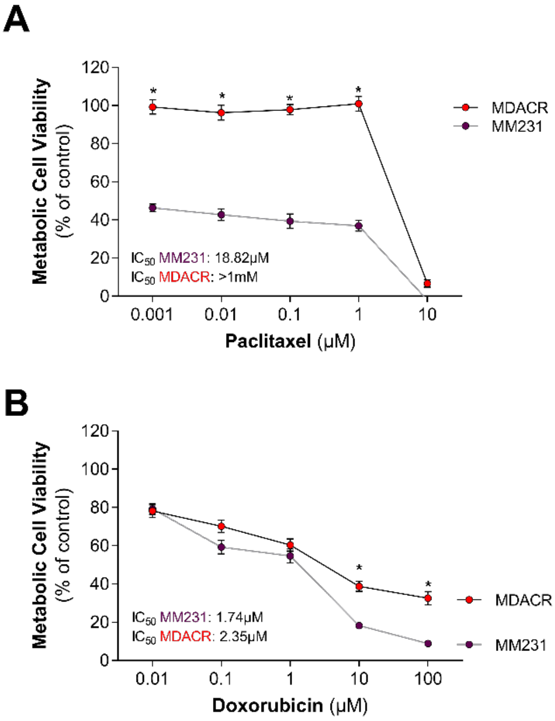

Figure S5: Analysis of cross-resistance to paclitaxel and doxorubicin in MDA-MB-231 and MDACR cells. MDA-MB-231 (MM231) and MDACR cell metabolic viability following treatment with (A) paclitaxel (concentration 0.001 – 10 µM) and (B) doxorubicin (0.1 -100µM) for 48h. IC_{50} values calculated by nonlinear regression of log(inhibitor) vs. response using least squares as fitting method in a 4 parameters calculation with variable slope. IC_{50} represents loss of 50% cell viability. Results are expressed as percentage of control (untreated cells) for each cell line. Data are mean ± SEM of at least three independent experiments performed in quadruplicate. *p <0.05 vs MM231 by two-way ANOVA followed by Bonferroni's post hoc test.

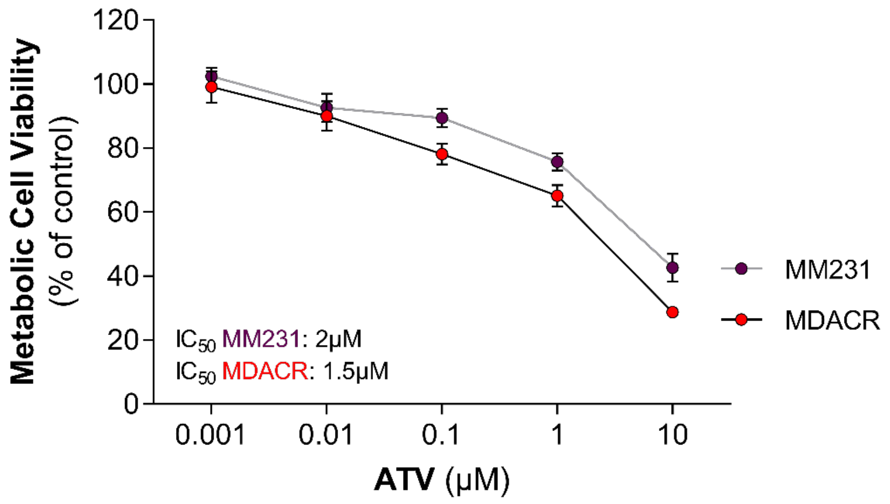

Figure S6: Comparison of ATV sensitivity in MDA-MB-231 and MDACR cells.
MDA-MB-231 (MM231) and MDACR cell metabolic viability following treatment with ATV (0.001 - 10 µM) for 48h. IC_{50} values calculated by nonlinear regression of log(inhibitor) vs. response using least squares as fitting method in a 4 parameters calculation with variable slope. IC_{50} represents loss of 50% of viability. Results are expressed as percentage of control (untreated cells) for each cell line. Data are mean ± SEM of three independent experiments performed in quadruplicate.

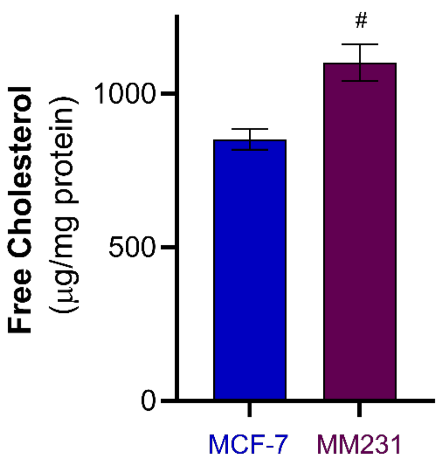

Figure S7: Intracellular free cholesterol content in MCF-7 and MDA-MB-231 cells.
MCF-7 and MDA-MB-231 (MM231) cell cholesterol content. Data are mean ± SEM of at least three independent experiments performed in triplicates. #$p < 0.05$ vs MCF-7 by two-tailed unpaired Student's t-test.

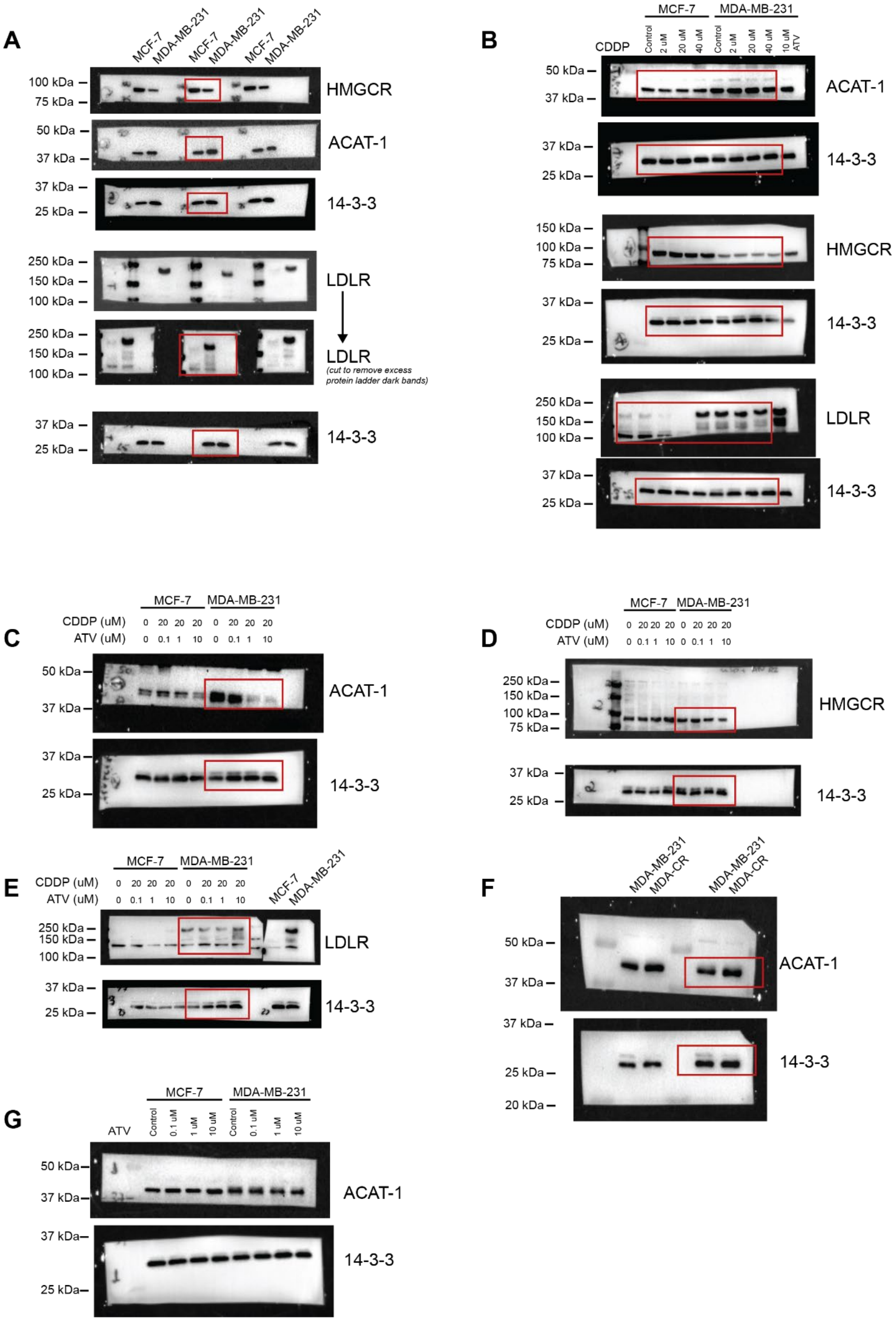

Figure S8: Immunoblots used as representative blots assembled in various figures of the manuscript. (A) and (B) show the full immunblot used to assemble Figure 5A and 5B, respectively. (C), (D) and (E) show the full immunblot used to assemble Figure 6C, 6D and 6E, respectively. (F) shows the full immunblot used to assemble Figure 7B. (G) show the full immunblot used to assemble Figure S3. All other figures were detected using ECL and Bio-Rad ChemiDoc System. Red boxes indicate the cropped portion of each immunoblot shown in the corresponding main figures.
